# Sarcopenia is associated with chemoradiotherapy discontinuation and reduced progression-free survival in glioblastoma patients

**DOI:** 10.1007/s00066-024-02225-7

**Published:** 2024-03-28

**Authors:** Fabian M. Troschel, Benjamin O. Troschel, Maren Kloss, Johanna Jost, Niklas B. Pepper, Amelie S. Völk-Troschel, Rainer G. Wiewrodt, Walter Stummer, Dorothee Wiewrodt, Hans Theodor Eich

**Affiliations:** 1 Department of Radiation Oncology, Münster University Hospital, Albert-Schweitzer-Campus 1, 48149 Münster, Germany; 2 Department of Neurosurgery, Münster University Hospital, Albert-Schweitzer-Campus 1, 48149 Münster, Germany; 3 Department of Medicine II, Klinikum Wolfsburg, Sauerbruchstraße 7, 38440 Wolfsburg, Germany; 4 Pulmonary Research Division, Münster University, Albert-Schweitzer-Campus 1, 48149 Münster, Germany; 5 Department of Pulmonary Medicine, Mathias Foundation, Hospitals Rheine and Ibbenbüren, Frankenburgsstraße 31, 48431 Rheine, Germany

**Keywords:** Palliative care, Body composition, Radiation therapy, Frailty, Prognostic biomarker, Computed tomography

## Abstract

**Purpose:**

Sarcopenia may complicate treatment in cancer patients. Herein, we assessed whether sarcopenia measurements derived from radiation planning computed tomography (CT) were associated with complications and tumor progression during radiochemotherapy for glioblastoma.

**Methods:**

Consecutive patients undergoing radiotherapy planning for glioblastoma between 2010 and 2021 were analyzed. Retrocervical muscle cross-sectional area (CSA) was measured via threshold-based semi-automated radiation planning CT analysis. Patients in the lowest sex-specific quartile of muscle measurements were defined as sarcopenic. We abstracted treatment characteristics and tumor progression from the medical records and performed uni- and multivariable time-to-event analyses.

**Results:**

We included 363 patients in our cohort (41.6% female, median age 63 years, median time to progression 7.7 months). Sarcopenic patients were less likely to receive chemotherapy (*p* < 0.001) and more likely to be treated with hypofractionated radiotherapy (*p* = 0.005). Despite abbreviated treatment, they more often discontinued radiotherapy (*p* = 0.023) and were more frequently prescribed corticosteroids (*p* = 0.014). After treatment, they were more often transferred to inpatient palliative care treatment (*p* = 0.035). Finally, progression-free survival was substantially shorter in sarcopenic patients in univariable (median 5.1 vs. 8.4 months, *p* < 0.001) and multivariable modeling (hazard ratio 0.61 [confidence interval 0.46–0.81], *p* = 0.001).

**Conclusion:**

Sarcopenia is a strong risk factor for treatment discontinuation and reduced progression-free survival in glioblastoma patients. We propose that sarcopenic patients should receive intensified supportive care during radiotherapy and during follow-up as well as expedited access to palliative care.

**Supplementary Information:**

The online version of this article (10.1007/s00066-024-02225-7) contains supplementary material, which is available to authorized users.

## Introduction

Glioblastoma patients face a limited prognosis despite trimodal therapy regimens (surgical resection followed by chemoradiotherapy) [1, 2]. Early progression is common and median overall survival is 14 months [1]. However, within the cohort, individual prognosis differs significantly [3]. While some patients are transferred to inpatient palliative care weeks after diagnosis, others may survive for years without tumor recurrence [3]. Treatment tolerance similarly varies, with some patients progressing during radiotherapy and/or discontinuing treatment due to deteriorating clinical condition [4].

Sarcopenia refers to reduced skeletal muscle strength or mass and is associated with decreased physical functioning [5]. Underlying mechanisms include aging, malnutrition, and systemic inflammation [6]. In cancer patients, sarcopenia may result in increased treatment- and cancer-related complications [7] as well as deteriorating outcomes [8, 9]. Assessment of sarcopenia on computed tomography (CT) imaging is a key noninvasive approach to identify this at-risk group [10].

We previously established a semi-automated threshold-based algorithm for muscle measurement on cranial glioblastoma radiation planning CT scans [11]. Patients with low muscle measurements showed reduced overall survival (OS) [11]. In this follow-up analysis in a larger cohort, we aimed to assess whether CT-based measurements are helpful to identify patients with more immediate clinically meaningful outcomes, including treatment-related complications and early cancer progression.

## Methods

The ethics committee of the Medical Association of Westphalia-Lippe (2021-685-f-S) approved this retrospective analysis.

### Patient cohort

We screened consecutive adult patients undergoing radiotherapy planning CT scans for histologically proven primary glioblastoma (isocitrate dehydrogenase 1 [*IDH1*] wildtype) at our institution between January 1, 2010, and December 31, 2021. Patients with incomplete clinical data and those with incomplete visualization of the C1 vertebra (either due to cutoff or due to streak artifacts) were excluded. We also omitted patients with prior cranial radiotherapy. Only patients with a novel glioblastoma diagnosis were considered, while recurrences were not included. Hence, inclusion criteria were similar to before [11] and the prior cohort from the exploratory study was part of this follow-up analysis. A screening flowchart is demonstrated in Supplementary Fig. 1.

Glioblastoma was defined according to the current definition of the World Health Organization (WHO) [12]. Clinical data collected included patient demographics (age, sex, height, weight, comorbidities according to the Charlson Comorbidity Index [CCI], and postoperative Eastern Cooperative Oncology Group [ECOG] score), tumor characteristics (date of diagnosis, tumor volume, O6-methylguanine-DNA methyltransferase [*MGMT*] promotor methylation, and *IDH1 *mutational status), and course of treatment (surgery date, resection status, planned radiotherapy dose and fractionation, applied radiotherapy dose and fractionation, chemotherapy application, prescription of corticosteroids, posttreatment stay, and progression during chemoradiotherapy). Hypofractionation was defined as a daily radiation dose exceeding 2 Gy. ECOG score was determined postoperatively at the onset of chemoradiotherapy, as recommended [13]. We quantified tumor volume on preoperative contrast-enhanced T1-weighted magnetic resonance imaging (MRI). Finally, we also collected data on first-time postoperative progression according to the Response Assessment in Neuro-Oncology Criteria (RANO) criteria [14]. Progression was defined as the time interval from diagnosis to MRI-based progression or death, whichever occurred first, similar to previous studies [15]. Data were partly abstracted from the patient records as well as from a neurosurgical database and the cancer registry of the Western German Cancer Center (*Westdeutsches Tumorzentrum*, WTZ).

### Muscle measurements

Non-contrast radiation planning CT scans were downloaded from the ARIA Oncology Information System (Varian Medical Systems, Inc., Palo Alto, USA). After upload onto 3D Slicer (version 4.13.0), we reformatted the CT scans along the frontal and vertical plains to ensure consistent measurements independent of patient positioning. This way, the nasal septum and the dens axis were both oriented in a vertical plain [11].

**Fig. 1:**
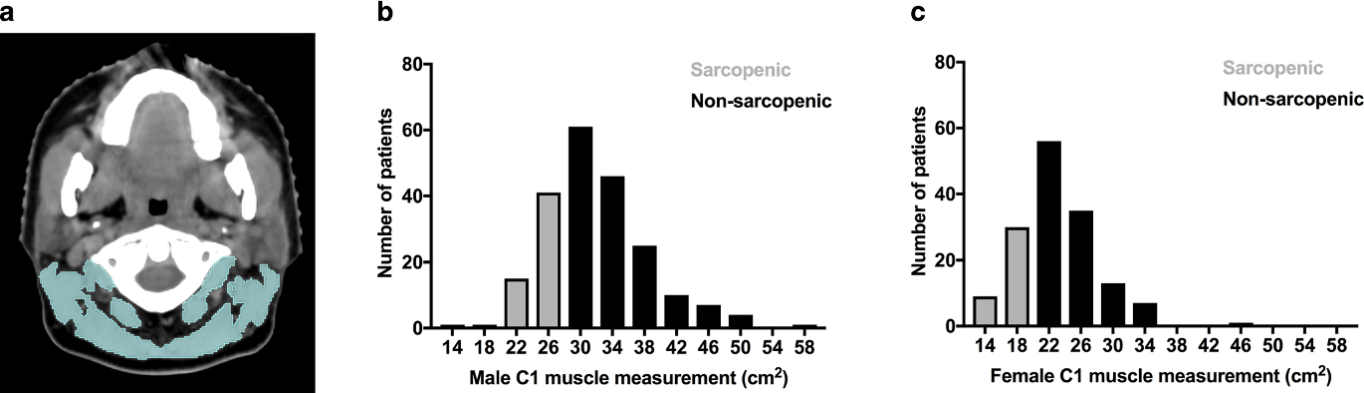
Body composition measurement results. **a** Illustration of the measurements on an axial cranial CT image at the height of the first cervical vertebra (C1). The retrocervical muscle area is marked in blue and encompasses the autochthonous, the trapezoid, the sternocleidomastoid and the levator scapulae muscles. **b**, **c**: Histograms of muscle measurements in male (**b**) and female (**c**) patients. Females had lower measurements compared to male patients. Patients in the lowest quartile of their sex were defined as sarcopenic, as marked in grey in the histogram

We measured the retrocervical muscle area on a single axial CT image at the level of the first cervical vertebra (C1), as previously established (Fig. 1a; [11]). Threshold-based semi-automated segmentations were performed with established cutoff values of −29 to +150 Hounsfield units [9]. Besides the autochthonous muscles, we also included the trapezoid, the sternocleidomastoid, and the levator scapulae muscles in the analyses. Quantifications resulted in a muscle cross-sectional area value, measured in square centimeters.

### Statistical analysis

Descriptive statistics were used to analyze patient characteristics. We compared the incidence of bivariate outcomes between sarcopenic and non-sarcopenic patients using the chi^2^ test. For progression-free survival (PFS), we chose a time-to-event analysis, starting at the time of histopathologic diagnosis. In univariable analyses, we used the Kaplan–Meier method and ran a log-rank test to evaluate statistical significance. In multivariable analyses, Cox proportional hazard regressions were applied. The same model previously developed for OS [11] was used for consistency for PFS and OS. Additionally, we generated a second multivariable PFS model restricted to patients who completed standard chemoradiotherapy (e.g., 60 Gy of radiation with concomitant chemotherapy). Statistics were performed using the STATA software package (version 13.0, StataCorp, College Station, TX). A *p*-value below 0.05 was considered significant.

## Results

### Patient cohort and treatment

We screened 372 glioblastoma patients treated at our institution and included 363 in the study (Supplementary Fig. 1). Median age was 63 years, median body mass index (BMI) was 26 kg/m^2^, and most patients were male (58.4%). Median CCI was 4, indicative of a low comorbidity score. Roughly half of the tumors showed *MGMT* methylation. Most glioblastomas were fully (44.6%) or partially (40.2%) resected, while the remainder (15.2%) underwent biopsy only (Table 1).

**Table 1:** Patient characteristics of the entire population (*n* = 363) and of the sarcopenic (*n* = 91) and non-sarcopenic groups (*n* = 272)

	All (*n* = 363)	Sarcopenic group (*n* = 91)	Non-sarcopenic group (*n* = 272)	*p*-value
*Age, years, median (range)*	63 (18–89)	69 (40–89)	61 (18–85)	< 0.001*
*Body mass index, kg/m*^*2*^*, median (range)*	26.0 (13.7–58.8)	24.9 (13.7–37.5)	26.5 (17.8–58.8)	0.006*
*Height, cm, median (range)*	173 (148–198)	172 (150–190)	174 (148–198)	0.14
*Weight, kg, median (range)*	80 (43–188)	77 (43–112)	80 (48–188)	0.001*
*Male, n (%)*	212 (58.4)	53 (58.2)	159 (58.5)	0.97
*Charlson Comorbidity Index, median (range)*	4 (0–12)	5 (0–12)	4 (0–12)	< 0.001*
*Eastern Cooperative Oncology Group (EGOG) score, n (%)*				< 0.001*
ECOG 0–1	260 (71.6)	50 (55.0)	210 (77.2)	
ECOG 2–4	103 (28.4)	41 (45.1)	62 (22.8)	
*MGMT methylation status, n (%)*				0.88
Unmethylated	169 (46.6)	43 (47.3)	126 (46.3)	
Methylated	194 (53.4)	48 (52.8)	146 (53.7)	
*Extent of tumor resection based on postoperative MRI, n (%)*				0.36
Gross total resection; > 95% resected	162 (44.6)	38 (41.8)	124 (45.6)	
Subtotal resection; > 5% and ≤ 95% resected	146 (40.2)	42 (46.2)	104 (38.2)	
Biopsy	55 (15.2)	11 (12.1)	44 (16.2)	
*Laterality of tumor manifestation, n (%)*				0.67
Unilateral	312 (86.0)	77 (84.6)	235 (86.4)	
Bilateral	51 (14.1)	14 (15.4)	37 (13.6)	
*Number of lesions at time of diagnosis, n (%)*				0.76
Single lesion	275 (75.8)	70 (76.9)	205 (75.4)	
Multiple lesions	88 (24.2)	21 (23.1)	67 (24.6)	
*Tumor size at time of diagnosis, cm*^*3*^	30.0 (0.3–170.9)	33.8 (3.4–163.5)	28.4 (0.3–170.9)	0.023*
*Tumor size postoperatively, cm*^*3*^*, in case of incomplete resection or biopsy*	5.5 (0.2–107.9) (*n* = 201)	6.9 (0.3–107.9) (*n* = 53)	4.73 (0.2–89.2) (*n* = 148)	0.21

Differences in patient characteristics between these two groups were compared using Mann Whitney U tests and chi-square tests, as appropriate. Tumor size was segmented based on contrast enhancing lesions on T1-weighted MRI scans; the laterality and number of lesions was assessed similarly

*Statistically significant *p*-value

Of 363 patients, 311 (85.7%) received the standard radiation dose of 60 Gy. 90.1% of patients were administered concomitant chemotherapy. Chemotherapy overwhelmingly included temozolomide according to Stupp et al. [16] (81.9%), or temozolomide and CCNU according to Herrlinger et al. [17] (14.1%). 52.6% of patients were prescribed corticosteroids during radiotherapy. 9.4% discontinued radiation. Overall, 303/363 (83.5%) of patients completed chemoradiotherapy (i.e., radiotherapy to a dose of 60 Gy and concomitant chemotherapy). 7.7% progressed during radiotherapy. After treatment completion, 86.3% continued outpatient care while 6.3% were transferred to inpatient palliative care treatment. Median PFS was 7.7 months (95% confidence interval 7.0–8.5 months). 89.5% of patients progressed during follow-up: 259 (71.3%) patients showed progression on follow-up imaging while 66 (18.2%) of patients died with no available prior evidence of progression. Finally, 38 (10.5%) patients did not progress during follow-up.

### Muscle measurements

Median muscle measurement was 27.7 cm^2^ (interquartile range 22.5–33.0 cm^2^). Men demonstrated a larger muscle area at 31.1 cm^2^ (interquartile range 27.6–35.2 cm^2^) compared to females at 22.2 cm^2^ (interquartile range 19.9–25.4 cm^2^; *p* < 0.001). Measurements of less than 27.6 cm^2^ in males and less than 19.9 cm^2^ in females were considered sarcopenic as they were in the lowest quartile of their peers (Fig. 1b, c). In sum, 91 patients (38 females and 53 males) were considered sarcopenic while 272 patients (113 females and 159 males) were considered non-sarcopenic.

Sarcopenic patients had a lower BMI (*p* = 0.006), were older (*p* < 0.001), and had higher CCI (*p* < 0.001) and ECOG scores (*p* < 0.001). Finally, tumors were larger at the time of diagnosis (*p* = 0.023). There were no differences in tumor characteristics otherwise, and resection status was not different between groups (Table 1).

### Radiochemotherapy treatment

**Fig. 2:**
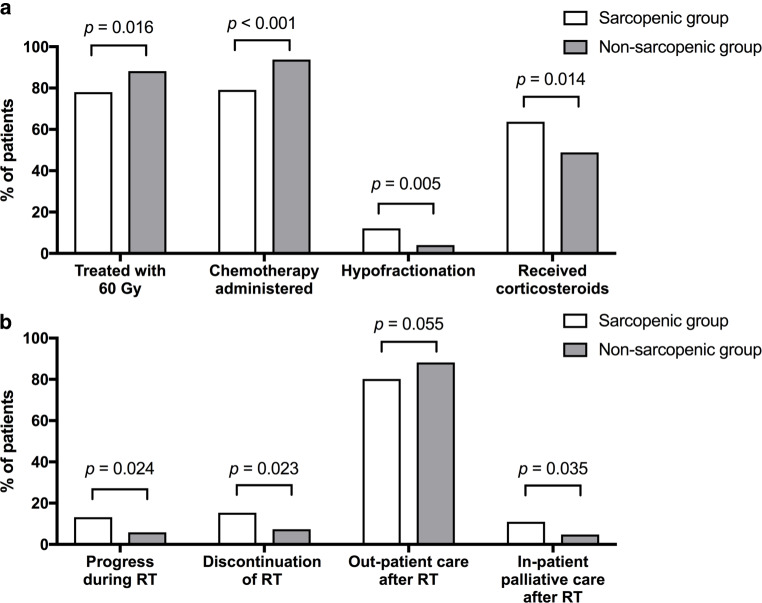
Differences in treatment application and tolerance between sarcopenic and non-sarcopenic patients. **a** Sarcopenic patients were less likely to receive the standard dose of 60 Gy and concomitant chemotherapy treatment. They were more likely to be treated with hypofractionated radiotherapy and more likely to be prescribed corticosteroids. **b** Sarcopenic patients more often progressed during therapy and discontinued treatment. They were less likely to be discharged home after treatment, but more likely to be transferred to in-patient palliative care

Sarcopenic patients were more infrequently treated with a standard dose of 60 Gy (78.0% vs. 88.2% for non-sarcopenic patients, *p* = 0.016). Concomitant chemotherapy was also more rarely administered (79.1% vs. 93.7%, *p* < 0.001). Conversely, radiotherapy was more often applied in a hypofractionated treatment regimen (12.1% vs. 4.0%, *p* = 0.005, Fig. 2a).

During treatment, sarcopenic patients were more often prescribed corticosteroids due to intracranial pressure symptoms (63.7% vs. 48.9%, *p* = 0.014). Sarcopenic patients showed a higher rate of progression during therapy (13.2% vs. 5.9%, *p* = 0.024) and radiotherapy was more often discontinued before reaching the prescribed dose (15.4% vs. 7.4%, *p* = 0.023). In sum, 72.5% of sarcopenic patients received standard chemoradiotherapy (i.e., radiation to a dose of 60 Gy in 2‑Gy fractions with concomitant chemotherapy), compared to 87.1% of non-sarcopenic patients (*p* = 0.001). Among those patients treated with chemotherapy, there was no difference in the choice of agent (temozolomide, temozolomide plus CCNU, or other) between the groups (*p* = 0.2, Supplementary Table 1). 11.0% of sarcopenic and 4.8% of non-sarcopenic patients were transferred to inpatient palliative care (*p* = 0.035). Conversely, sarcopenic patients were less likely to remain in outpatient care (as opposed to inpatient treatment) after completion of radiotherapy (80.2% vs. 88.2%, *p* = 0.055, Fig. 2b).

### Progression-free survival

**Fig. 3:**
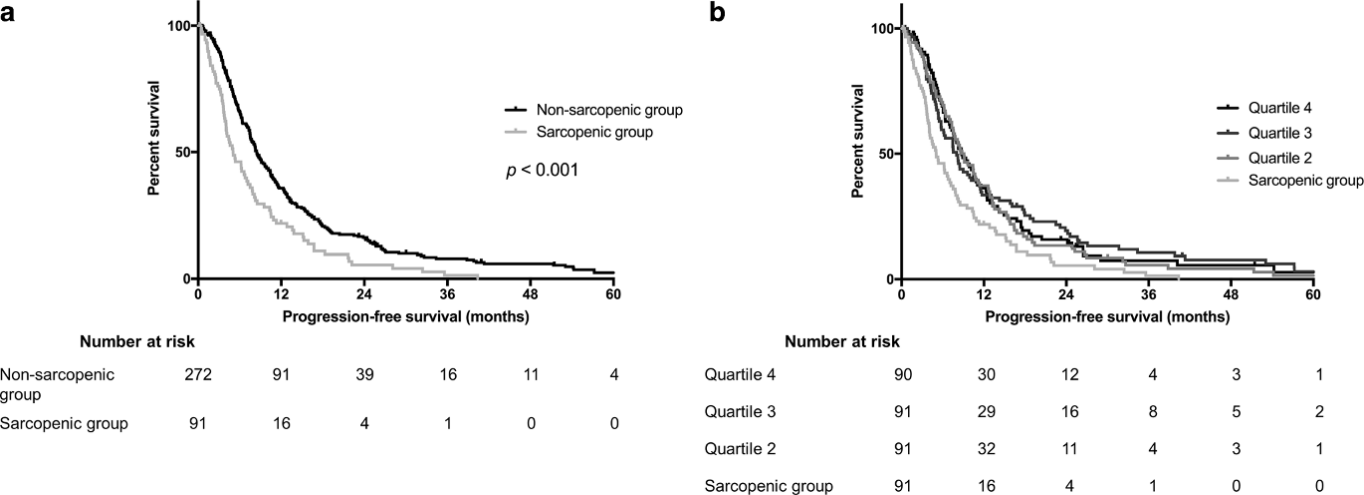
Progression-free survival (PFS) in sarcopenic and non-sarcopenic patients in univariable analyses. **a** Sarcopenic patients showed a reduced PFS compared to non-sarcopenic patients. **b** There was no difference in PFS within the non-sarcopenic group (e.g., between patients in the second, third, and fourth quartile of muscle measurements)

PFS was markedly reduced in sarcopenic patients in univariable analyses in Kaplan–Meier assessment (Fig. 3a) and log rank test (*p* < 0.001). Median time to progression was 5.1 months (95% confidence interval 4.0–7.0 months) in sarcopenic patients and 8.4 months in the non-sarcopenic group (95% confidence interval 7.5–9.9 months). When assessing all muscle measurement quartiles separately, no significant differences in PFS were seen between the upper three quartiles (median time to progression was 9.1, 8.0, and 8.7 months for the second, third, and fourth quartile, respectively; Fig. 3b). We then performed multivariable modeling, describing hazard ratios (HR) and confidence intervals (CI) for the association between patient, tumor, or treatment parameters (including sarcopenia measurements) and PFS (Table 2). When including predefined risk factors, sarcopenia remained independently associated with PFS (HR 0.61 [CI 0.46–0.81] relative to non-sarcopenic patients, *p* = 0.001). Additionally, BMI, CCI, and bilateral tumors showed a negative, while extent of resection and methylated *MGMT* status showed a positive association with PFS (Table 2). We also tested continuous muscle measurements in a multivariable model, finding that increased muscle measurements (per cm^2^) were independently associated with prolonged PFS (HR 0.96 [CI 0.94–0.99], *p* = 0.002, Table 2). In a final analysis, we restricted the multivariable model to patients completing standard chemoradiotherapy treatment (i.e., treatment with 60 Gy in 2 Gy fractions and concomitant chemotherapy) to assess independence of muscle-related associations from treatment completion. The significance of muscle measurements remained unchanged both in analyses for sarcopenic/non-sarcopenic groups (HR 0.65 [CI 0.47–0.89], *p* = 0.008) and for continuous measurements (HR 0.97 [CI 0.94–0.99], *p* = 0.007) in this subgroup (Supplementary Table 2).

**Table 2:** Multivariable Cox proportional hazard regression of progression-free survival (PFS) in glioblastoma patients

	Model A		Model B	
	HR (CI)	*p*-value	HR (CI)	*p*-value
*Muscle measurement*				
Model A: non-sarcopenic group vs. sarcopenic group	0.61 (0.46–0.81)	0.001*	–	–
Model B: C1 muscle area (cm^2^)	–	–	0.96 (0.94–0.99)	0.002*
*Body mass index, kg/m*^*2*^	1.04 (1.02–1.06)	< 0.001*	1.05 (1.02–1.07)	< 0.001*
*Sex*				
Female	Ref. 1.00	–	Ref. 1.00	–
Male	0.98 (0.78–1.24)	0.87	1.36 (0.99–1.87)	0.058
*Charlson Comorbidity Index, points*	1.09 (1.02–1.17)	0.01*	1.09 (1.02–1.17)	0.009*
*ECOG score*				
0–1	Ref. 1.00	–	Ref. 1.00	–
≥ 2	1.26 (0.96–1.64)	0.095	1.24 (0.95–1.62)	0.11
*Tumor size at time of diagnosis, cm*^*3*^	1.00 (0.99–1.00)	0.11	1.00 (0.99–1.00)	0.12
*Number of lesions at time of diagnosis*				
1	Ref. 1.00	–	Ref. 1.00	–
2 or more	1.27 (0.96–1.67)	0.096	1.24 (0.94–1.64)	0.13
*Tumor extent*				
Unilateral	Ref. 1.00	–	Ref. 1.00	–
Bilateral	2.68 (1.87–3.84)	< 0.001*	2.74 (1.91–3.93)	< 0.001*
*MGMT methylation status*				
Unmethylated	Ref. 1.00	–	Ref. 1.00	–
Methylated	0.57 (0.45–0.72)	< 0.001*	0.58 (0.46–0.72)	< 0.001*
*Extent of tumor resection based on postoperative MRI, n (%)*				
Gross total resection; > 95% resected	Ref. 1.00	–	Ref. 1.00	–
Subtotal resection; > 5% and ≤ 95% resected	1.27 (0.99–1.62)	0.06	1.27 (0.99–1.63)	0.056
Biopsy	2.05 (1.41–2.98)	< 0.001*	2.12 (1.45–3.08)	< 0.001*

PFS was defined as the interval from first diagnosis to progression or death, whichever occurred first. Tumor size was segmented based on contrast-enhancing lesions on T1-weighted MRI scans; the laterality and number of lesions was assessed similarly. The Charlson Comorbidity Index (CCI) is as a combination score of age and comorbidities. Two models for different muscle measures were calculated in different models: model A (assessing dichotomized muscle measurements as sarcopenic or non-sarcopenic), and model B (assessing continuous muscle measurements at the level of the first cervical vertebra in cm^2^). Analyses were performed in *n* = 363 patients

*Statistically significant *p*-value

*CI* confidence interval, *ECOG* Eastern Cooperative Oncology Group, *HR* hazard ratio, *MGMT* O6-methylguanine-DNA methyltransferase, *Ref.* Reference (reference group in group-based comparisons)

Our novel sarcopenia definition was also strongly associated with OS in univariable log-rank analyses (median survival 9.1 vs 15.2 months, *p* < 0.001, Supplementary Fig. 2) as well as in multivariable modeling (HR 0.64, CI 0.49–0.85, *p* = 0.002, Supplementary Table 3). This extends and reconfirms previous exploratory results [11].

## Discussion

In this study, we found that sarcopenia is a strong risk factor for reduced treatment regimens, treatment discontinuation, and diminished PFS in glioblastoma patients. We suggest that sarcopenic patients may profit from enhanced follow-up and expedited palliative care access.

Our work is based on a prior proof-of-concept study in glioblastoma patients. It demonstrated that radiation planning CTs could be used for body composition measures at C1, that these measures were representative of whole-body measurements, and that muscle measures were associated with OS in glioblastoma patients [11].

In this follow-up study in an enlarged and updated patient cohort, we investigated whether body composition parameters were clinically valuable for identifying patients at risk for adverse treatment courses. Here, we a priori defined patients in the lowest quartile of muscle measurements as sarcopenic. While predefined cutoffs for sarcopenic patients are available at multiple levels in the trunk [10], no data exist regarding the neck. Subsequently, we defined a cohort-based cutoff, similar to previous studies [18]. Based on this setup, we drew multiple key conclusions, which are discussed in the following sections.

### Sarcopenic patients undergo abbreviated postoperative treatment regimens

The American Society for Radiation Oncology (ASTRO) guideline [19] suggests age-stratified glioblastoma treatment with hypofractionated radiotherapy recommended for patients older than 70 years. The guideline’s authors note that age cutoff values may differ substantially between studies and many subgroup analyses are small and retrospective in nature [19]. Age also remains an imperfect variable given variation in physical functioning, quality of life, and prognosis between patients of similar age [20, 21]. Elderly patients with good physical functioning may indeed profit from chemoradiotherapy as opposed to radiotherapy alone [22]. Thus, the authors also suggest stratifying by “performance status” to better individualize treatment recommendations [19]. However, Karnofsky index and ECOG score are prone to high interrater variability [23, 24].

As a quantitative, non-rater-dependent biomarker, sarcopenia measurements incorporate both age and performance status [25]. Sarcopenia becomes more prevalent with age [25] and is negatively associated with physical functioning [5]. Some studies refer to body composition parameters as measures of “biologic age” as they encompass nutritional factors, functioning, inflammation, and chronological age [26]. This is reflected in our findings, as sarcopenia was associated with these factors in our cohort: Significant associations were found for chronological age, BMI, comorbidities, and ECOG score. The strongest signal was found for ECOG, demonstrating a close association between sarcopenia and (reduced) physical functioning, as expected [27]. Notably, whether sarcopenia is the result of a limited physical status or vice versa remains unclear, as the retrospective nature of our study allows for tests regarding association, but not causation. In any case, sarcopenia is a quantitative marker related to numerous otherwise difficult-to-quantify parameters.

Importantly, as discussed above, sarcopenia is associated with key treatment stratification parameters from the ASTRO guideline. Consistent with this, sarcopenic patients were less likely to receive the standard chemoradiotherapy treatment and more likely to undergo hypofractionated irradiation without chemotherapy in our study. Interestingly, despite abbreviated and de-escalated treatment prescription, patients still discontinued radiotherapy at a higher rate. This underlines the need for reduced treatment paradigms in sarcopenic patients. Hence, sarcopenia may be a potential prospective marker that helps guide treatment decision-making.

### Treatment tolerance is reduced in sarcopenic patients

It is well known that elderly patients are at risk for increased treatment toxicities, most prominently from chemotherapy [28]. Treating younger patients with poor performance status also remains a major concern, yet data are more limited [29].

Sarcopenic patients were more often prescribed corticosteroids during treatment (64% vs. 49%). Steroids may change body composition measures in the long term [30] and aggravate sarcopenia. However, this is unlikely to have affected our measurements, as radiation planning CTs are commonly performed within weeks after primary diagnosis and neurosurgical evaluation, and before application of radiotherapy. Our data indicate that sarcopenic patients may be more at risk for brain edema symptoms, as these trigger corticosteroid therapy during radiation.

While chemoradiotherapy discontinuation was increased among sarcopenic patients, 85% of sarcopenic patients still completed the prescribed therapy. We believe our data suggest that sarcopenic patients may profit from more intensive medical care during therapy. However, the relatively high completion rate of radiotherapy among sarcopenic patients may support continued use of postoperative radiotherapy (with the potential omission of chemotherapy) in this group, similar to findings from the ASTRO guideline for elderly patients or those with low performance status [19]. However, our study is retrospective in nature and this question may only be definitively answered in prospective trials.

Upon treatment completion, a small subpopulation of patients were directly transferred to in-patient palliative care, likely indicative of limited outcomes. While these patients were overrepresented among sarcopenic patients (11% vs. 5%), the vast majority of sarcopenic patients, 80%, returned home after treatment. Sarcopenia may nonetheless help to identify patients who could profit from intensified, and early, palliative care interventions.

### Progression-free survival is substantially reduced in sarcopenic patients

The associations between sarcopenia and OS [31, 32] or complications [33–35] have been well described in numerous malignancies. Most studies imply that reduced physical functioning (commonly associated with sarcopenia [5]) is likely to make patients more susceptible to developing complications, limiting survival [36].

In our study, sarcopenic glioblastoma patients were more likely to show early progression. This is an intriguing finding, as PFS (different from OS) is likely not directly associated with reduced physical functioning. We initially hypothesized that treatment discontinuation in sarcopenic patients may induce early progression, similar to other malignancies [37]. However, while treatment was more limited in some sarcopenic patients, nearly three quarters of sarcopenic patients received full standard chemoradiotherapy treatment. Thus, we considered differences in treatment completion or chemotherapy application as unlikely to account for the drastic difference in PFS across the entire sarcopenic population (median PFS 5.1 vs. 8.4 months). When assessing this hypothesis, we were reluctant to include chemoradiotherapy treatment completion in our multivariable PFS model as we aimed to only include parameters available at the onset of postoperative treatment. Chemotherapy discontinuation may be advised during treatment depending on blood test results [38]. Hence, we generated a second, separate multivariable model and only included patients who completed standard chemoradiotherapy. In this homogeneously treated cohort, the strong association between sarcopenia and PFS remained unchanged. Consequently, we concluded that the link between sarcopenia and PFS was not mediated via treatment discontinuation.

Similarly, sarcopenic patients did not show differences in tumor characteristics or resection status, making it unlikely that these factors confounded our findings. Notably, tumor volume at diagnosis was slightly increased in sarcopenic patients, raising questions regarding the interplay between increased tumor growth and a rising incidence of sarcopenia. This association has been confirmed in different tumor entities [39]. However, despite inclusion of tumor volume in our multivariable model, sarcopenia was independently associated with PFS. Interestingly, residual tumor volume was not significantly different between the groups after resection.

Thus, after careful consideration of potential clinical confounders, we believe that sarcopenia is instead likely to be associated with PFS via biological factors. In other tumor entities, these include sarcopenia-related immune senescence [40] and proinflammatory signaling [41]. In glioblastoma, immunosuppression increases treatment resistance [42, 43] and induces a distinct neuroinflammatory microenvironment that promotes tumor growth and invasion [44]. In lung cancer, immunotherapy (which is not part of standard treatment in glioblastoma patients) showed a substantially reduced efficacy in sarcopenic patients, potentially indicative of an altered baseline immune system in this group [45]. Prospective studies have linked sarcopenia to systemic inflammation and a distinct disbalance in the immune system [46].

We suspect that sarcopenic glioblastoma patients are a subgroup of patients with an unfavorable inflammatory and immune status. This may potentially promote tumor progression, leading to the reduction in PFS we see in the sarcopenic subgroup.

Clinically, this finding is meaningful, as the optimal follow-up intervals for glioblastoma patients remain unclear [47]. In the absence of specific evidence, MRIs are commonly scheduled every 12 weeks following intra-institutional pragmatic considerations [47, 48]. Novel computational studies try to predict individualized time to progression to optimize this decision [49]. Here, sarcopenia may be a valuable parameter. Our study indicates that it may be reasonable for sarcopenic patients to undergo more frequent follow-up imaging, e.g., every 8 weeks. However, given moderate tolerance of primary treatment and aggressive tumor growth in sarcopenic patients, the clinical benefit of sometimes intensive retreatment [50] in this vulnerable subgroup might be debatable and should be considered with care. Notably, our measurements—which become available only postsurgically—are neither designed nor intended to change the role of up-front surgical resection, which remains the gold standard for virtually all glioblastoma patients.

As expected, based on the previous exploratory study [11], our increased cohort again shows a convincing association with overall survival, both in univariable and multivariable modeling. Different from the previous study, we used our novel sarcopenia definition in this analysis (as opposed to simply dividing a cohort by the median, as done in the exploratory stage). The strong difference in median survival of roughly 6 months demonstrates that our sarcopenic measurements have identified an at-risk cohort with good selectivity.

CT-based measurements are universally attainable in radiotherapy patients. The diversification of the glioma diagnosis based on methylation profiling and gene expression [51, 52] will increase the need for commonly available parameters to identify populations in which treatment escalation or de-escalation is advisable. This was also reflected in a recent post-hoc analysis of the CATNON trial [53], questioning the concurrent use of TMZ in adjuvant glioblastoma radiotherapy, and the lack of a clear treatment standard for patients with relapsed disease. Identifying sarcopenia early might help to guide treatment decisions in different prognostic groups and stages of treatment. End-to-end pipelines for automation of measurements have been built and may facilitate clinical implementation [54]. Automated scoring results (e.g., percentiles based on the overall patient cohort) may then be considered for therapeutic decision-making. However, prospective data collection and validation needs to precede any assessment of clinical application.

Sarcopenia may be modifiable to some degree, mainly through changes in nutrition and physical activity [55]. However, whether these interventions improve outcomes remains unclear. Our study indicates that patients with very low muscle measurements would potentially profit most from interventions. However, these patients may suffer from neurologic symptoms, such as hemiplegia, limiting the potential for physical exercise. Nonetheless, we believe that past and ongoing physical exercise [56–58] and nutritional investigations [59] in glioblastoma patients have merit.

Some study limitations should be noted. First, this is a retrospective monocentric analysis with the corresponding risk of bias. However, we included a large cohort of patients and patient characteristics were largely representative of the overall glioblastoma population. Nonetheless, we remain unable to address prospective questions such as the modifiability of sarcopenia. Second, multivariable modeling was not possible for treatment application and tolerance outcomes due to high treatment adherence and low complication rates. However, multivariable modeling was performed for PFS. Third, PFS was not available in all patients. However, a large majority of patients, more than 70%, had available PFS. Fourth, semi-automated segmentations required some manual correction, potentially limiting clinical application. However, body composition measurements have been shown to be automatable thanks to novel computational methods [60]. Finally, MRI-based radiation planning may alleviate the need for cranial planning CTs in the future, preventing opportunistic sarcopenia measurements. However, while technically more challenging, sarcopenia measures are also feasible on MRI imaging [61].

## Conclusion

In conclusion, we found that sarcopenia is a strong risk factor for abbreviated treatment regimens, treatment discontinuation, and reduced progression-free survival in glioblastoma patients. As a distinct at-risk group, sarcopenic patients may profit from intensified care during treatment, enhanced follow-up after treatment completion, and expedited access to palliative care. Prospective trials are needed to further investigate the potential relevance of sarcopenia for therapeutic decision-making.

### Supplementary Information

Supplementary Figure 1: Inclusion and exclusion criteria for the study population. Supplementary Figure 2: Overall survival (OS) in sarcopenic and non-sarcopenic patients in univariable analyses. Supplementary Table 1: Choice of agent among patients treated with concomitant chemotherapy. Supplementary Table 2: Multivariable Cox proportional hazard regression of progression-free survival (PFS) in glioblastoma patients, restricted to patients that completed standard chemoradiotherapy. Supplementary Table 3: Multivariable Cox proportional hazard regression of overall survival (OS) in glioblastoma patients
