# Supplementary material for: Sarcopenia is associated with chemoradiotherapy discontinuation and reduced progression-free survival in glioblastoma patients
Source: Strahlenther Onkol. 2024 Mar 28;200(9):774–84. doi: 10.1007/s00066-024-02225-7 (PMC11343971; doi:10.1007/s00066-024-02225-7)
Supplement: Supplementary file 1 — Supplementary Figure 1: Inclusion and exclusion criteria for the study population. Supplementary Figure 2: Overall survival (OS) in sarcopenic and non-sarcopenic patients in univariable analyses. Supplementary Table 1: Choice of agent among patients treated with concomitant chemotherapy. Supplementary Table 2: Multivariable Cox proportional hazard regression of progression-free survival (PFS) in glioblastoma patients, restricted to patients that completed standard chemoradiotherapy. Supplementary Table 3: Multivariable Cox proportional hazard regression of overall survival (OS) in glioblastoma patients [file 66_2024_2225_MOESM1_ESM.pdf]

Supplementary Figure 1: **Inclusion and exclusion criteria for the study population.**

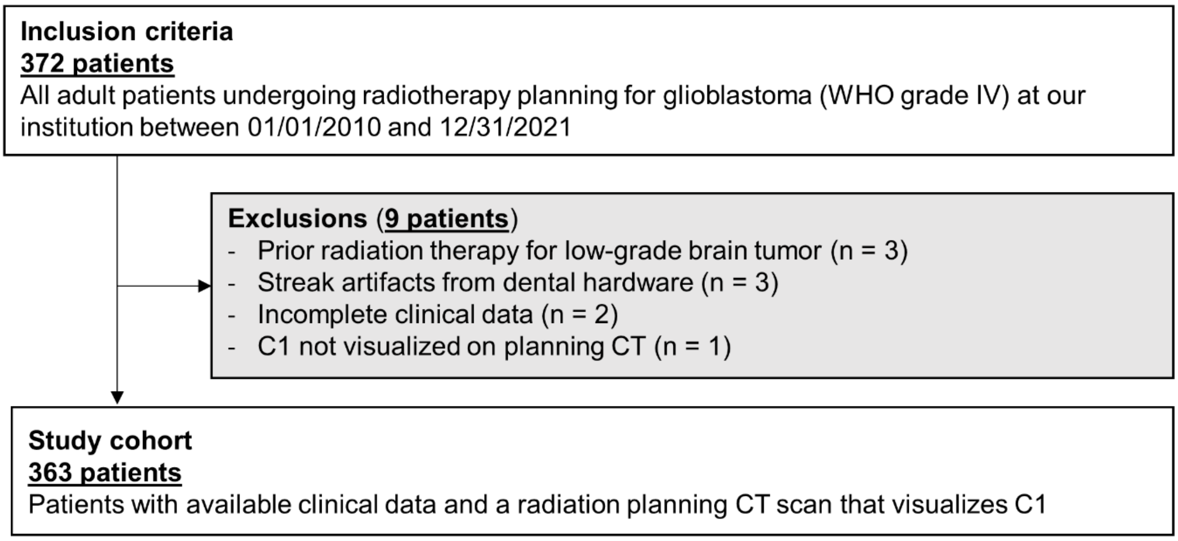

Supplementary Figure 2: Overall survival (OS) in sarcopenic and non-sarcopenic patients in univariable analyses. Sarcopenic patients showed a reduced OS compared to non-sarcopenic patients in log-rank analyses ($p < 0.001$) and Kaplan Meier plots.

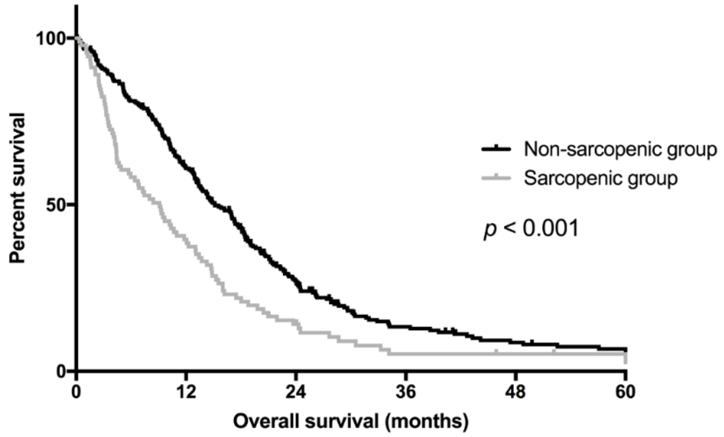

Number at risk

	0	12	24	36	48	60
Non-sarcopenic group	272	162	62	25	14	10
Sarcopenic group	91	36	12	4	3	2

Supplementary Table 1: **Choice of agent among patients treated with concomitant chemotherapy.** There was no difference between the sarcopenic and the non-sarcopenic group.

**Choice of agent among patients treated with concomitant chemotherapy**, n (%)	All (n = 363)	Sarcopenic group (n = 91)	Non-sarcopenic group (n = 272)	*p* value
Temozolomide (according to Stupp et al.)	269 (81.7)	63 (86.3)	206 (80.5)	0.20
Temozolomide and lomustine (CCNU) (according to Herrlinger et al.)	47 (14.3)	6 (8.2)	41 (16.0)	
Other	13 (4.0)	4 (5.5)	9 (3.5)	

Supplementary Table 2: **Multivariable Cox proportional hazard regression of progression-free survival (PFS) in glioblastoma patients, restricted to patients that completed standard chemoradiotherapy.** PFS was defined as the interval from first diagnosis to progression or death, whichever occurred first. Tumor size was segmented based on contrast enhancing lesions on T1-weighted MRI scans; the laterality and number of lesions was assessed similarly. The Charlson Comorbidity Index (CCI) is as a combination score of age and comorbidities. Two models for different muscle measures were calculated in different models, Model A (assessing dichotomized muscle measurements as sarcopenic or non-sarcopenic), and Model B (assessing continuous muscle measurements at the level of the first cervical vertebra in cm²). Standard chemoradiotherapy completion was defined as application of 60 Gy in 2 Gy fractions to the tumor region with concomitant application of chemotherapy. Analyses were performed in n = 303 patients.

	Sarcopenic group vs. Non-sarcopenic group		C1 muscle area (cm²)	
	HR (CI)	*p*	HR (CI)	*p*
**Muscle measurement**				
**Model A:** Non-sarcopenic group vs. sarcopenic group	0.65 (0.47-0.89)	**0.008**		
**Model B:** C1 muscle area (cm²)			0.97 (0.94-0.99)	**0.007**
**Body mass index**, kg/m²	1.04 (1.02-1.07)	**<0.001**	1.05 (1.03-1.08)	**<0.001**
**Sex**				
Female	Ref 1.00		Ref 1.00	
Male	1.10 (0.85-1.43)	0.45	1.52 (1.06-2.16)	**0.022**
**Charlson Comorbidity Index**, points	1.07 (0.98-1.16)	0.14	1.07 (0.98-1.16)	0.14
**ECOG Score**				
0-1	Ref 1.00		Ref 1.00	
≥2	1.22 (0.90-1.66)	0.19	1.20 (0.88-1.62)	0.25
**Tumor size at time of diagnosis,** cm³	1.00 (0.99-1.00)	0.082	1.00 (0.99-1.00)	0.078
**Number of lesions at time of diagnosis**				
1	Ref. 1.00		Ref 1.00	
2 or more	1.35 (0.99-1.85)	0.061	1.36 (0.99-1.86)	0.059
**Tumor extent**				
Unilateral	Ref 1.00		Ref 1.00	
Bilateral	3.20 (2.11-4.86)	**<0.001**	3.16 (2.08-4.81)	**<0.001**
**MGMT methylation status**				
Un-methylated	Ref 1.00		Ref 1.00	
Methylated	0.52 (0.40-0.67)	**<0.001**	0.53 (0.41-0.69)	**<0.001**
**Extent of resection**				
Gross total resection (GTR); >95% resected	Ref 1.00		Ref 1.00	
Subtotal resection (STR); >5% and <95% resected	1.20 (0.92-1.56)	0.19	1.21 (0.93-1.58)	0.16
Biopsy	1.78 (1.14-2.78)	**0.012**	1.83 (1.17-2.86)	**0.009**

CI: confidence interval. ECOG: Eastern Cooperative Oncology Group. HR: Hazard ratio. MGMT: O6-Methylguanine-DNA Methyltransferase. Ref.: Reference (reference group in group-based comparisons).

Supplementary Table 3: **Multivariable Cox proportional hazard regression of overall survival (OS) in glioblastoma patients.** Tumor size was segmented based on contrast enhancing lesions on T1-weighted MRI scans; the laterality and number of lesions was assessed similarly. The Charlson Comorbidity Index (CCI) is as a combination score of age and comorbidities. Analyses were performed in n = 363 patients.

	Sarcopenic group vs. Non-sarcopenic group	
	HR (CI)	*p*
**Muscle measurement**		
Sarcopenic group	Ref. 1.00	
Non-sarcopenic group	0.64 (0.49-0.85)	**0.002**
**Body mass index**, kg/m^2	1.04 (1.01-1.06)	**0.001**
**Sex**		
Female	Ref 1.00	
Male	1.16 (0.92-1.46)	0.22
**Charlson Comorbidity Index**, points	1.12 (1.05-1.19)	**<0.001**
**ECOG Score**		
0-1	Ref 1.00	
≥2	1.46 (1.12-1.89)	**0.004**
**Tumor size at time of diagnosis,** cm^3	1.00 (0.99-1.00)	0.54
**Number of lesions at time of diagnosis**		
1	Ref. 1.00	
2 or more	1.27 (0.95-1.70)	0.10
**Tumor extent**		
Unilateral	Ref 1.00	
Bilateral	2.01 (1.42-2.86)	**<0.001**
**MGMT methylation status**		
Un-methylated	Ref 1.00	
Methylated	0.60 (0.48-0.75)	**<0.001**
**Extent of resection**		
Gross total resection (GTR); >95% resected	Ref 1.00	
Subtotal resection (STR); >5% and <95% resected	1.21 (0.94-1.57)	0.14
Biopsy	2.33 (1.62-3.35)	**<0.001**

CI: confidence interval. ECOG: Eastern Cooperative Oncology Group. HR: Hazard ratio. MGMT: O6-Methylguanine-DNA Methyltransferase. Ref.: Reference (reference group in group-based comparisons).
